# The Influence of Thermal and Mechanical Stress on the Electrical Conductivity of ITO-Coated Polycarbonate Films

**DOI:** 10.3390/polym15112543

**Published:** 2023-05-31

**Authors:** Christopher Johannes, Fabian Lins, Miriam Meyer, Michael Hartung, Hans-Peter Heim

**Affiliations:** Institute of Material Engineering, Plastics Engineering, University of Kassel, 34125 Kassel, Germany

**Keywords:** ITO, electrochromic device, polycarbonate

## Abstract

The influence of thermomechanical stress on the conductivity of indium tin oxide (ITO)-coated polycarbonate (PC) films was investigated. PC is the industry’s standard material for window panes. ITO coatings on polyethylene terephthalate (PET) films are the main commercially available option; as such, most investigations refer to this combination. The investigations in this study aim to investigate the critical crack initiation strain at different temperatures and crack initiation temperatures for two different coating thicknesses and for a commercially available PET/ITO film for validation purposes. Additionally, the cyclic load was investigated. The results show the comparatively sensitive behavior of the PC/ITO films, with a crack initiation strain at room temperature of 0.3–0.4% and critical temperatures of 58 °C and 83 °C, with high variation depending on the film’s thickness. Under thermomechanical loading, the crack initiation strain decreases with increasing temperatures.

## 1. Introduction

Metal oxide layers are used in various applications, such as electrochromic systems, displays, and sensor technology [1,2,3,4,5]. The following metal oxides are often used as current collector layer in electrodes: fluorine-doped tin oxide (FTO), antimony-doped tin oxide (ATO), aluminum-doped tin oxide (AZO), and indium tin oxide (ITO), the last of which is used in most applications. The ITO coating investigated in this paper is generally characterized by high transparency with low electrical resistance (20–200 Ω/sq; 70–90% transmittance). In principle, coatings with a lower electrical sheet resistance can also be obtained, but such coatings exhibit slightly yellow discoloration and low transmittance as a result of the layer’s thickness [6].

The brittleness of ITO is one of the main problems when it is used in combination with flexible plastic substrates, as the conductivity can be significantly reduced by cracking due to mechanical and/or thermal stress [7,8,9,10]. Due to the different thermal expansion coefficients of the metal oxide layer and the plastic substrate, stresses occur during temperature variations that occur in later use, which can ultimately result in the formation of cracks in the layer [11,12,13].

The application of tensile stress causes a sudden increase in the electrical resistance of the ITO layers, which is often observed to occur between 1.3% and 1.4% strain in polyethylene terephthalate (PET) substrates. In this case, the cracks develop around existing defects in the layer and propagate from there across the complete width of the specimens [12,14,15,16]. The influence of the annealing temperature on the mechanical load capacity of ITO-coated PET substrates was investigated in a study by Machinaga et al. The mechanical strength of the ITO layer was measured using the electrical resistance. Here, the ITO layer showed a strong increase in electrical resistance caused by cracking above 2.8% strain. As the annealing temperature increases, this crack initiation strain also increases simultaneously. This indicates the significant dependency of the crack initiation strain on the annealing temperature [17].

The film’s thickness also has a significant influence on the critical strain, since the crack propagation depends on the corresponding strain energy, which is higher for a thicker layer for the same substrate stress. Compared to thin layers, thicker layers may have larger defects, resulting in lower strength. Additionally, it has been observed that, despite cracks appearing across the complete sample width, conductive paths between adjacent ITO fragments remain. It is believed that these conductive paths are located at the bottom of the crack. This theory is strengthened by the fact that the electrical resistivity is finite [14].

In the study by Saleh et al., PET/ITO samples were subjected to cyclic dynamic loading. It was found that after the formation of cracks and the associated increase in electrical resistance, a partial closing of the cracks can be observed when the load is removed. This leads to a decrease in electrical resistance [16]. In the cyclic tests conducted by Cairns et al., it was found that the resistance at loading levels below the critical strain was independent of the strain, while the resistance above the critical strain increased sharply; this can be attributed to crack initiation. Contrary to other studies, it was found that, after cracks occurred and the specimen was unloaded, the electrical resistance decreased only marginally compared to the initial resistance [18].

**Figure 1:**
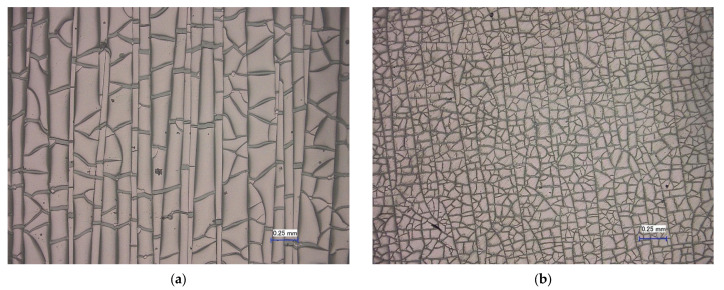
Microscope image of (**a**) compressed and (**b**) stretched ITO layers (radius 1000, 100× resolution). Reprinted with permission from Ref. [19].

In principle, a distinction must be made between the compression and stretching of the individual layers in the case of bending stress. If the ITO layer is compressed, delamination of the ITO layer from the substrate can occur (Figure 1a), while stretching leads to crack formation (Figure 1b) [10,18,19]. Compression only has a minor effect on the electrical resistance, since the crack flanks are adjacent to each other due to the compression [18].

It is assumed that crack initiation starts at the layer’s edges. The cracks run almost parallel to each other and orthogonal to the tensile stress. Furthermore, it is suspected that the cracks do not initiate at the surface of the ITO layer but in the interlayer of the ITO and the substrate. In addition to cracks, wrinkles appear in the ITO layer under bending; this is due to the shrinkage deformation caused by the Poisson effect [17,20,21].

The literature shows that, so far, investigations have mainly focused on ITO layers on PET substrates. In this study, the influence of thermomechanical stress on the conductivity of ITO-coated polycarbonate (PC) films was investigated. These are used by the authors to fabricate electrochromic films, which are intended to be back-molded in an injection molding process and further processed into compact electrochromic polycarbonate panes. Due to its very good optical and mechanical properties, PC is a preferred amorphous plastic for optical applications such as window panes or headlight covers. For example, both its impact strength and haze are significantly better than those of PET. The heat deflection temperature of 120 °C is also significantly higher than that of amorphous PET at 65 °C (HDT/A) [22]. The investigations conducted in this study aim to investigate the critical crack initiation temperature and crack initiation strain of ITO on PC films at different temperatures. It is to be verified that, with increasing temperatures, the crack initiation strain ε_crack_ decreases linearly; this is because, compared to the plastic substrate, the ITO has a much lower thermal expansion coefficient (7.6 × 10^−6^ K^−1^ [16]).

In order to show the dependency of the initial resistance of the layer and the initial crack resistance on the layer thickness, two different layer thicknesses were studied. In the cyclic tensile tests, the conductivity behavior below and above the crack initiation strain was investigated. In order to validate the measurement method, a commercially available PET/ITO film was investigated.

## 2. Materials and Methods

The ITO-coated PET film (127 µm) with an ITO layer thickness t of 130 nm (Sigma Aldrich Chemie GmbH, Taufkirchen, Germany) was used as purchased. The PC film Makrofol 1-1 (Covestro AG, Leverkusen, Germany) with a thickness of 250 µm was sputtered with two different commercially available ITO coatings, ‘Elamet Trans B’ and ‘Elamet Trans C’ from Nanogate GfO Systems GmbH (Schwäbisch Gmünd, Germany), which differ in terms of their layer thickness, sheet resistance, and transmittance. The substrate materials’ properties are summarized in Table 1 and the sheet resistance and transmittance at 550 nm of the ITO-coated substrates are given in Table 2.

**Figure 2:**
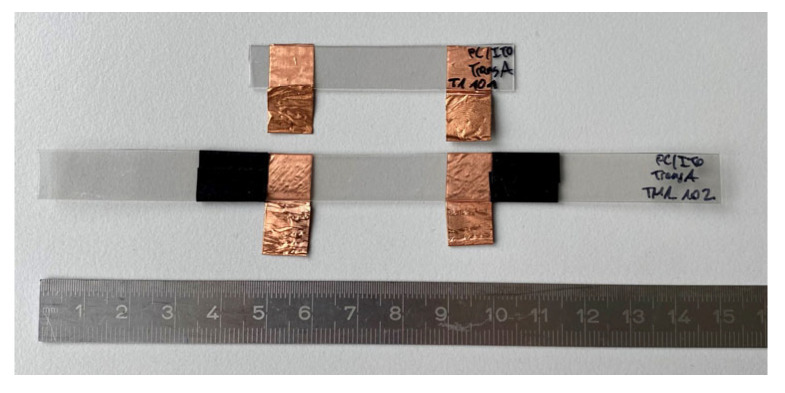
Sample geometry for thermal stress (top) and tensile, cyclic, and thermo-mechanical tests (bottom).

In accordance with the sample geometry from Saleh et al. [16], the geometry of Figure 2 was used. Due to the different testing methods, two types of samples were used. The larger sample had a length of 150 mm and a width of 10 mm and was used for the (thermo-) mechanical tests. For the thermal tests, the specimen length was reduced to 60 mm to minimize material consumption. The distance between the contact points for measuring the electrical resistance was 30 mm for all samples. The distance for determining the strain using an extensometer was 50 mm.

The samples were cut using a scalpel, and a copper tape (AT526, 10 mm × 0.035 mm, Advance Tapes International Ltd., Thurmaston, UK) was applied to produce more conductive bonding points. Conditioning was carried out in a climatic test chamber 3433 (Feutron, Langenwetzendorf, Germany) for 24 h at a humidity of 50% and a temperature of 23 °C.

In all tests, the electrical resistance of the layer was recorded as an indicator for the integrity of the conductive layer. The resistance was measured using a digital multimeter HMC8012 (Rohde & Schwarz, Munich, Germany), and 4-channel measurement was used to exclude measurement errors due to the heating of the cables. Normalized resistance was used for the evaluation, with *R*_0_ recorded at 0.05% strain. Equation (1) shows the normalized resistance. A 10% increase in electrical resistance compared to the initial resistance is judged to be significant and has also been applied in various studies [1,23]:

(1) $$R^{*}=\frac{R}{R_{0}}$$

Thermal stress was applied using a temperature chamber of a universal testing machine 101 (Zwick Roell, Ulm, Germany). The samples were heated from 25 °C to 100 °C at a heating rate of 5 K/min. The electrical resistance was recorded as a function of temperature.

**Figure 3:**
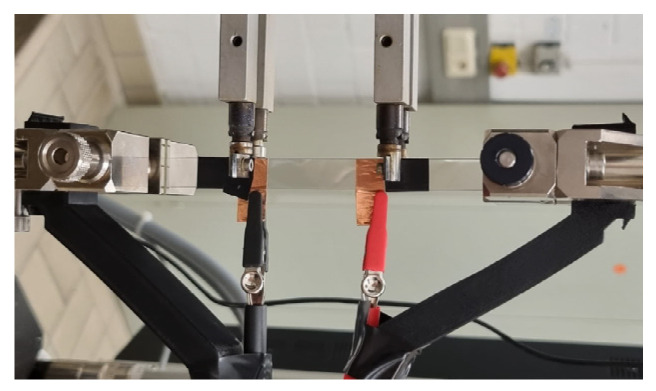
Experimental setup for thermal, mechanical, and thermo-mechanical tests.

The experimental setup was identical for the tensile, cyclic, and thermomechanical testing (Figure 3). The tests were carried out on a universal testing machine 101 (Zwick Roell, Ulm, Germany). A load cell (F_max_ = 1000 N) and sample grips were used for the films. The samples were tested at a test speed of 1 mm/min, which is recommended in the DIN ISO 178 for corresponding films. The samples were clamped into the fixture and first fixed on one side. Subsequently, the contacting clamps for the resistance measurement were connected to the respective contact points. In order to reduce the influence of the clamps’ weight, 3D printed holders were used.

For the cyclic tensile test, five different strain levels (0.2%, 0.4%, 0.6%, 0.8%, and 1.0%) were used. After reaching the first elongation level, the sample was unloaded to a force of 1 N and, subsequently, the next elongation level was approached. A spectroscopic ellipsometer (J.A. Woollam, Lincoln, NE, USA) with a wavelength range of 190–2400 nm was used to determine the layer thicknesses. The values of the amplitude ratio ψ and phase shift Δ were fitted using the Cauchy model. Three measurements were conducted for each coating type.

## 3. Results and Discussion

**Figure 4:**
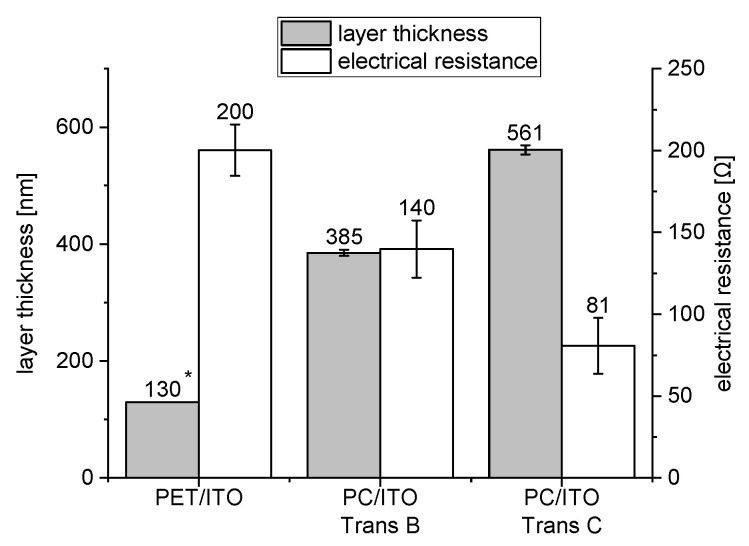
Electrical resistance (*n* = 5) and layer thickness (*n* = 3) of the different ITO layers before mechanical testing. Error bar equals two standard deviations; * according to datasheet.

The ITO layer’s thicknesses and electrical resistances are depicted in Figure 4. The results confirm that the initial electrical resistance decreases as the ITO layer’s thickness increases, with the PC/ITO Trans C layer (561 nm, 81 Ω) having the lowest initial resistance and highest layer thickness, the PET/ITO layer (130 nm, 200 Ω) having the highest initial resistance and lowest layer thickness, and PC/ITO Trans B (385 nm, 140 Ω) being in between. As mentioned in the introduction, the crack initiation strains of the various ITO layers are decisively influenced by the layer thickness.

**Figure 5:**
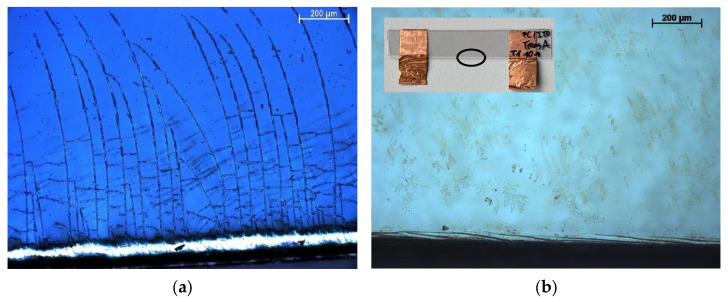
Cracks in the (**a**) PC/ITO (385 nm) and (**b**) PET/ITO (130 nm) layer at the edge of the sample after cutting.

Regardless of the stress type, the edge areas of the various layers exhibit characteristic crack structures due to the cutting process (Figure 5). In the PC/ITO (385 nm) samples, the cracks run from the edge region about one millimeter into the interior of the layer (Figure 5a); meanwhile, in the PET/ITO (130 nm) samples, the cracks run largely parallel to the edge and extend only about 0.1 mm into the interior (Figure 5b). This crack structure can be explained by the elastic and plastic deformation that occurs during the cutting process, which causes the brittle ITO layer to crack. The crack tips in the edge areas are particularly problematic under the thermal and/or mechanical stress investigated here, since stress peaks in the layer occur at these points and cracks might propagate from there over the entire surface [21].

**Figure 6:**
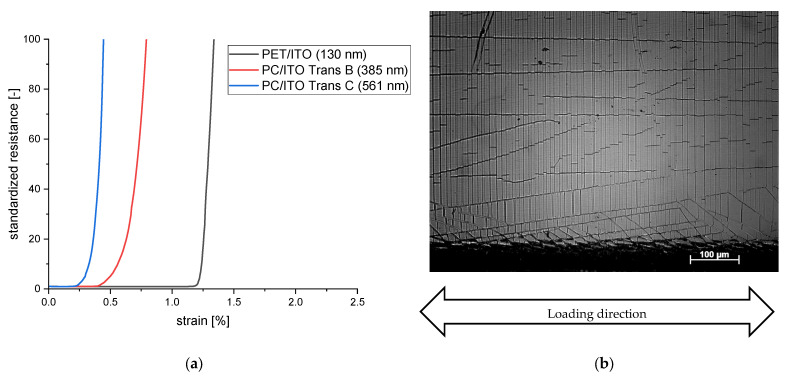
(**a**) Course of the normalized resistance of ITO layers under mechanical strain (*n* = 1). (**b**) Cracks in the PC/ITO layer after applying mechanical strain.

The typical course of the normalized resistance of the ITO layer under mechanical strain is almost constant until the critical strain is reached, and then it increases sharply (Figure 6a). There is a certain initial crack strain at which the mechanical stress on the ITO layer exceeds a threshold value, causing the layer to fail mechanically and cracks to form; this result is similar to that reported in the literature [11,12]. These cracks lead to a sharp increase in electrical resistance since the air between the cracks has an insulating effect. Figure 6b shows a pronounced crack structure orthogonal to the loading direction after the tensile test, as well as wrinkles that are presumably due to the Poisson effect in the tensile direction.

**Figure 7:**
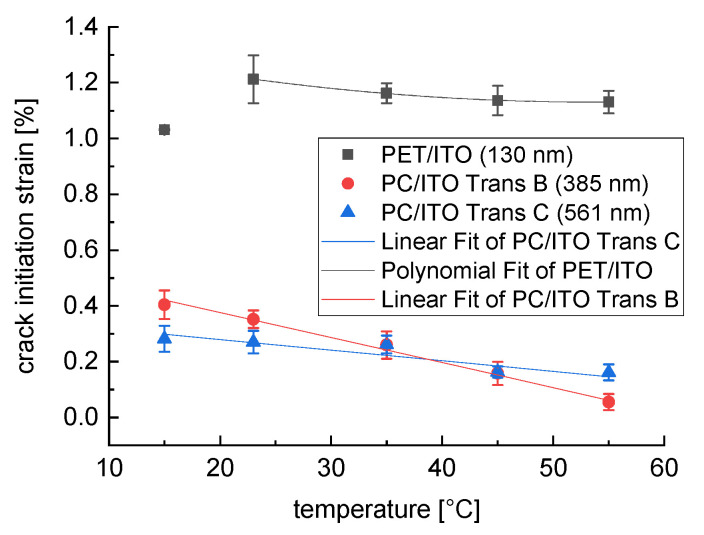
Temperature-dependent crack initiation strain of PC/ITO Trans B, PC/ITO Trans C, and PET/ITO (*n* = 3), error bar equals two standard deviations.

**Figure 8:**
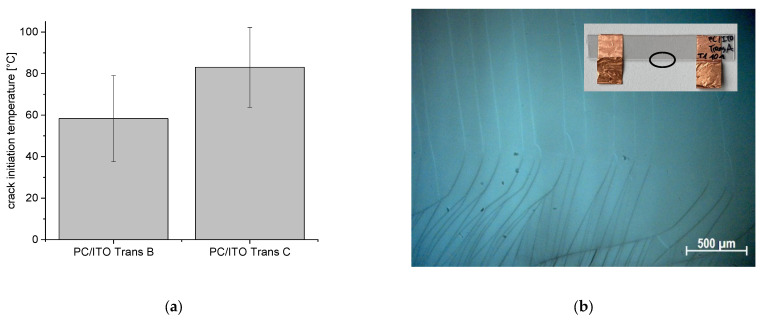
(**a**) Crack initiation temperatures of PC/ITO Trans B (*n* = 10) and PC/ITO Trans C (*n* = 5); error bar equals two standard deviations. (**b**) Crack development in the PC/ITO layer under thermal stress from the edge area (bottom) through the specimen width (top).

The PET/ITO layer has the highest crack initiation strain at room temperature, with ε(23 °C) = 1.21% on average (Figure 7); this concurs fairly well with the values in the literature and therefore validates the measuring method [16]. The crack initiation strain of the PC/ITO Trans C has the lowest value with ε(23 °C) = 0.27%, while the PC/ITO Trans B is more resistant with ε(23 °C) = 0.35% on average. This result supports the hypothesis that the layer thickness has a decisive influence on the crack initiation strain, what is also supported by various studies [14,15,16]. The significantly lower level of the crack initiation strain of the PC/ITO may also be related to the more pronounced crack structure (Figure 5a), since the stress cracks apparently start precisely at these crack tips (Figure 8b). Comparative measurements with specimens sputtered only after cutting could provide further information on this phenomenon.

Figure 7 shows the crack initiation strain of the different ITO layers at five different temperatures between 15 °C and 55 °C. It can be observed that, regardless of the ITO type, additional thermal loading reduces the crack initiation strain; this supports the hypothesis that the higher thermal expansion coefficient of the plastic substrate (80 × 10^−6^ K^−1^) compared to that of ITO (7.6 × 10^−6^ K^−1^) leads to thermally induced stress in the ITO layer. For the experiments conducted at 15 °C, the experimental chamber was cooled down using liquid nitrogen. The results of the two PC/ITO types show a consistently higher crack initiation strain at 15 °C, compared to 23 °C for PC/ITO Trans B (ε(15 °C) = 0.40%) and PC/ITO Trans C (ε(15 °C) = 0.28%), whereas a lower crack initiation strain was measured for the PET/ITO samples (ε(15 °C) = 1.03%). A possible explanation for this is that, due to the shrinkage of the substrate, a kind of wrinkling with simultaneous delamination of the ITO layer might occur, perhaps fostered by microcracks that are already present in the ITO layer. This behavior was previously observed by Kim et al. [17]. The temperature at which the ITO layer was sputtered onto the substrates is unknown, but this information might be relevant in this context.

While the crack initiation strains of PC/ITO Trans B and PC/ITO Trans C show essentially linear temperature dependence, as expected, this seems to be nonlinear for PET/ITO (the value at 15 °C was excluded in the polynomial fit of the data). This behavior could indicate the presence of relaxation effects, which are amplified by the raised test temperatures. Here, the thermally and mechanically induced stresses in the interface layer might have been reduced due to relaxation, considering the aforementioned low heat deflection temperature of PET, at 65 °C. Nevertheless, the mean deviations are not statistically significant, so further investigations must be carried out.

The data suggest the more sensitive behavior of the PC/ITO Trans B layer compared to the PC/ITO Trans C layer. This result indicates that the different thicknesses of the Trans B (385 nm) and Trans C (561 nm) ITO layers may have an effect on the sensitivity. Meanwhile, it was expected that the resistance would be higher with a thinner ITO layer, considering the results for PET/ITO (130 nm) with significantly higher crack initiation strains. The substrate material, as well as the sputter material and parameters, might have an important influence on the crack initiation strain. However, the design of this study does not permit any substantiated statements to be made in this regard.

Thermal stressing of the PET/ITO samples shows no increase in resistance up to 100 °C, while the PC/ITO samples show similar sharp increases in electrical resistance and in mechanical stressing, indicating rapid crack propagation and/or increasing crack density in the layer. The formation of such cracks running through the entire sample, starting from the existing cracks in the edge region (introduced during sample preparation), was confirmed by microscopic examination (Figure 8b). The crack initiation temperatures are 58 °C and 83 °C for PC/ITO Trans B and PC/ITO Trans C, respectively (Figure 8a), and fit well with the curves in Figure 7 when extrapolated. Both results have high variance, which could originate from the size and density of the initial cracks due to sample preparation. Furthermore, in the case of thermal stress, a biaxial stress is present in the sample; meanwhile, in the case of tensile stress, it is only uniaxial.

**Figure 9:**
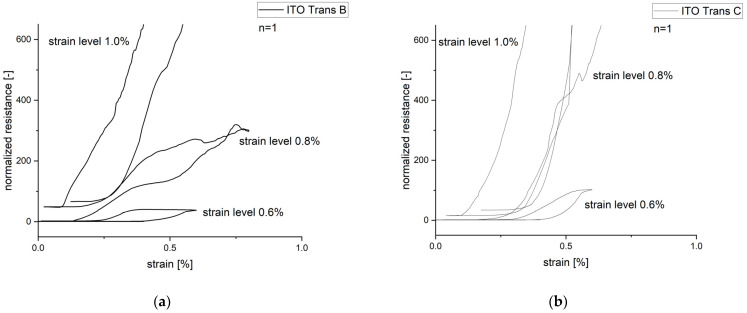
Course of normalized resistance under cyclic loading for (**a**) PC/ITO Trans B and (**b**) PC/ITO Trans C; the specimen was pulled sequentially to 5 different strains (0.2%, 0.4%, 0.6%, 0.8%, and 1.0%), with unloading after each strain level.

The following section discusses the behavior of the normalized resistance of the PC/ITO samples under cyclic loading, which is shown in Figure 9. For both the PC/ITO Trans B and Trans C coating types, no changes in the normalized electrical resistance occurred at strain level 1 (ε = 0.2%), which is below the crack initiation strains at 23 °C, and even at level 2 (ε = 0.4%), which is slightly higher than the average crack initiation strains at 23 °C. This result was not expected. On the one hand, it is possible that the measured samples had a crack initiation strain above 0.4%, which is quite plausible considering the scatter of the measured values at 23 °C (Figure 7); on the other hand, the preceding level 1 strain could have had an influence. An increase in resistance occurred for both coating types at the third strain level (ε = 0.6%). The level of resistance at the different strain levels differs depending on the coating type. Furthermore, the resistance drops close to the initial resistance after the third strain cycle, while a higher final resistance is present in the subsequent strain cycles. This behavior can be explained by the elastic and plastic deformation of the PC substrate. A predominant elastic deformation causes the cracks to close partially or completely while relieving, causing the resistance to drop to almost the initial value as a result. The higher the plastic deformation part is, the more cracks exist that do not close while relieving, and a higher resistance remains. The thicker ITO Trans C coating differs mainly in terms of the slope of the resistance increase, which rises more rapidly (Figure 9b). This behavior can be explained by the higher brittleness of the Trans C coating due to its greater coating thickness. This results in a higher crack density due to the mechanical strain, which leads to a more rapid increase in resistance.

## 4. Conclusions and Outlook

The results of the investigation of the PC/ITO films confirm the well-known behaviors of PET/ITO films, whereby cracks form in the conductive ITO layer as a result of both thermal and mechanical stress, which leads to a significant increase in the electrical resistance. The two investigated ITO layer types, Trans B (385 nm, 140 Ω) and Trans C (561 nm, 81 Ω), differ in terms of their layer thickness and thus their electrical resistance and transmittance. At room temperature, the thicker layer shows a lower crack initiation strain (ε(23 °C) = 0.27%) than the thinner one (ε(23 °C) = 0.35%), as expected, but both are much more sensitive than the commercially available PET/ITO film (130 nm, 200 Ω) with ε(23 °C) = 1.21%, which was used to validate the measurement method. The main reasons for the large difference are likely to be the layer thickness and the much more pronounced cracks at the edge of the PC/ITO caused by cutting the specimen, as these are the starting point for crack growth under loads. Other possible influencing factors could be the ITO material, the sputtering parameters, and the substrate—it could be interesting to investigate these factors in another, more comprehensive study. In the PC/ITO samples, the crack initiation strain exhibits a linear dependency on the temperature. Here, the crack initiation strain decreases due to the additional thermally induced stress, as expected. Interestingly, the thinner ITO layer is more sensitive than the thicker layer at higher temperatures, as is reflected by a lower crack initiation strain at 55 °C (ε(55 °C) = 0.06%) and a crack initiation temperature of 58 °C on average, compared to values of ε(55 °C) = 0.16% and 83 °C for the thicker layer. The results of the cyclic test confirm the phenomenon that cracks close again when the specimens are unloaded or the crack flanks touch, leading to a significantly lower resistance level compared to the loading situation, up to the initial resistance. Considering the very high sensitivity of the investigated ITO layers on PC compared to the widely used PET/ITO substrates, they do not seem suitable for use in flexible EC films and for further processing in injection molding processes, given the high temperatures and pressures that occur in these scenarios. Promising alternative flexible conductors, such as metallic mesh structures with additional PEDOT:PSS coatings, graphene, carbon nanotubes, or Ag nanowires, each have advantages and disadvantages [23]. Purely polymeric conductors, which mainly comprise coatings of PEDOT:PSS, enable completely metal(-oxide)-free systems whose layers can potentially all be applied in large-scale manufacturing processes such as slot-die coating, and which exhibit excellent flexibility [24,25]. The challenge here is to ensure that the layer’s conductivity reaches the same level as that of the ITO while maintaining high transmittance.

## Figures and Tables

**Table 1:** Properties of the substrate materials [22].

Properties	PC Film	PET Film	Unit
Density	1.2	1.3–1.4	g/cm^3^
Yield stress	60–68	55	MPa
Yield strain	5.5–6.5	4	%
Elastic modulus	2350–2400	2100–2400	MPa
Heat deflection temperature (HDT/A)	120–130	60–65	°C
Thermal expansion coefficient	65–70	80	10^−6^/K

**Table 2:** Properties of the ITO layers according to datasheets.

Substrate Material	Material	Sheet Resistance (Ω/sq)	Transmittance at 550 nm (%)
PC	ITO (Elamet Trans B)	25–35	≥80
PC	ITO (Elamet Trans C)	15–20	≥70
PET	ITO (Sigma Aldrich)	45–65	≥78

## Data Availability

The data are available upon the reasonable request from the corresponding author.
